# Non-invasive hemodynamic profiling of patients undergoing hemodialysis - a multicenter observational cohort study

**DOI:** 10.1186/s12882-019-1542-4

**Published:** 2019-09-03

**Authors:** Keren Doenyas-Barak, Marcia H. F. G. de Abreu, Lucas E. Borges, Helcio A. Tavares Filho, Feng Yunlin, Zou Yurong, Nathan W. Levin, Allen M. Kaufman, Shay Efrati, David Pereg, Ilya Litovchik, Shmuel Fuchs, Sa’ar Minha

**Affiliations:** 1 Nephrology Department Shamir Medical Center (Assaf-Harofeh campus), Zeriffin, Israel; 2 Sackler School of Medicine, Tel-Aviv University, Ramat-Aviv, Israel; 3 Biocor Hospital de Doenças Cardiovasculares, Belo Horizonte, Brazil; 4 Renal Division, Sichuan Academy of Medical Sciences and Sichuan Provincial People’s Hospital, Chengdu, China; 5 Mt. Sinai School of Medicine, New York, NY USA; 6 Dialyze Direct, Brooklyn, NY USA; 7 Cardiology Department Meir Medical Center, Kfar-Saba, Israel; 8 Cardiology Department Shamir Medical Center (Assaf-Harofeh campus), Zeriffin, Israel

**Keywords:** Intradialytic hypotension, Hemodynamics, Non-invasive monitoring

## Abstract

**Background:**

Intradialytic blood pressure (BP) measurement is currently the main parameter used for monitoring hemodynamics during hemodialysis (HD). Since BP is dependent on cardiac output and total peripheral resistance, knowledge of these parameters throughout the HD treatment would potentially be valuable.

**Methods:**

The use of a novel non-invasive monitoring system for profiling hemodynamic response patterns during HD was explored: a whole-body bio-impedance system was used to assess cardiac index (CI), total peripheral resistance index (TPRI), cardiac power index (CPI) among other parameters in chronic HD patients from 4 medical centers. Measurements were made pre, during and post dialysis. Patients were grouped into 5 hemodynamic profiles based on their main hemodynamic response during dialysis i.e. high TPRI; high CPI; low CPI; low TPRI and those with normal hemodynamics. Comparisons were made between the groups for baseline characteristics and 1-year mortality.

**Results:**

In 144 patients with mean age of 67.3 ± 12.1 years pre-dialysis hemodynamic measurements were within normal limits in 35.4% but only 6.9% overall remained hemodynamically stable during dialysis. Intradialytic BP decreased in 65 (45.1%) in whom, low CPI (47 (72.3%)) and low TPRI (18 (27.7%) were recorded. At 1-year follow-up, mortality rates were highest in patients with low CPI (23.4%) and low TPRI (22.2%).

**Conclusions:**

Non-invasive assessment of patients’ response to HD provides relevant hemodynamic information that exceeds that provided by currently used BP measurements. Use of these online analyses could potentially improve the safety and performance standards of dialysis by guiding appropriate interventions, particularly in responding to hypertension and hypotension.

**Electronic supplementary material:**

The online version of this article (10.1186/s12882-019-1542-4) contains supplementary material, which is available to authorized users.

## Background

Hemodialysis (HD) induces significant hemodynamic imbalances due to rapid intravascular volume reduction, fluid and electrolyte shift, often occur simultaneously. This imposes significant stress on the heart and peripheral vasculature and leads to activation of various compensatory mechanisms necessary for the preservation of tissue perfusion. Since a large proportion of HD patients have coexisting cardiovascular comorbidities, their ability to compensate for these changes may be hampered by reduced cardiac output (CO) at baseline, autonomic neuropathy, or the concomitant use of drugs. While some patients are able to compensate adequately for blood pressure reductions, a substantial fraction of treatments will be accompanied by intradialytic hypotension (IDH), which is associated with poor long term outcomes [1].

At present, blood pressure (BP) measurement is the pivotal objective intradialytic monitoring parameter.. Pre-dialysis BP together with patient weights and clinical examination are used to guide dry weight goals and ultrafiltration rate. However, pre-dialytic low BP and/or the rapid development of IDH will require changes in dialysis plan or a modification of the patient’s chronic drug prescriptions. Currently, these modifications are largely based on clinical judgment in the absence of objective hemodynamic data.

BP is determined and affected three components- heart rate (HR), stroke volume (SV) and systemic vascular resistance. Scarce data exists regarding the hemodynamic changes induced by dialysis. This is probably due to the need for invasive methods (e.g. pulmonary artery catheter) to asses these parameters. Conflicting reports exist regarding the physiological responses to HD with most data coming from small scale studies and obsolete HD protocols [2–7]. More recently, continuous hemodynamic assessment has become feasible with the emergence of non-invasive monitoring systems. The objective of this study was to explore and describe the various hemodynamic changes that occur during chronic HD utilizing such a device.

## Methods

### Patients and data collection

This was a prospective multi-center cohort study performed in 4 medical centers (Queens Artificial Kidney Unit, New York, United States of America, Sichuan Provincial People’s Hospital, Chengdu, China, Assaf-Harofeh Medical Center, Zerifin, Israel and Hospital de Doenças Cardiovasculares, Belo Horizonte, Brazil). The data were collected between 8/2015 and 8/2017. The study protocol was approved by the local institutional review board in each center and all patients had signed an informed consent form. Included were patients older than 18 years that had undergone chronic hemodialysis for at least 3 months. A trained technician was available for a time period in each center and after obtaining consent, assisted in performing the monitoring per protocol. The number of included patients in each center was determined by the allocated time available for this technician. Beyond age and dialysis chronicity, no inclusion nor exclusion criteria existed.

Baseline characteristics including age, sex, weight, height, body mass index (BMI), dialysis vintage and diabetes mellitus status were collected prospectively. Dialysis prescription data included the duration of dialysis, total fluid removed and ultrafiltration rate (expressed as liter of fluid removed per kilogram weight per hour). All-cause mortality was assessed in all patients within 12 ± 1 months after the monitored session.

### NICaS hemodynamic monitoring

NICaS (NI Medical, Petah-Tikva, Israel) is a commercially available whole-body impedance based hemodynamic analysis system [8]. Bioimpedance measurements during the cardiac cycle allow the calculation of SV and together with HR, the calculation of CO. Measurements are adjusted to body surface area to yield stroke index (SI) and cardiac index (CI). Mean arterial pressure (MAP; calculated from standard blood pressure measurements) together with CI allows the calculation of total peripheral resistance index (TPRI = MAP/CI*80) and cardiac power index (CPI = MAP*CI/451 W/m2). TPRI represents the resistance to flow. It expresses the interplay between the cardiac index and the pressure gradient across the vascular tree (between the left ventricle and the right atrium). Low TPRI may reflect low blood pressure or high cardiac index while high TPRI indicates an opposite trend. CPI represents the heart’s ability to deliver hydraulic energy needed for adequate peripheral perfusion. This important metric is independently associated with adverse outcome in heart failure patients [9–11]. Collectively, these measurements allow a full, online representation and analysis of all the relevant components associated with specific hemodynamic status.

CI measurements obtained by this device correlate well with both pulmonary artery catheterization and echocardiography in acute heart failure patients [8, 12, 13]. An excellent correlation with echocardiography was also demonstrated during HD [14]. A recent study utilizing this device demonstrated the hemodynamic characteristics of IDH episodes [15].

### Hemodynamic profiling definitions

**Fig. 1:**
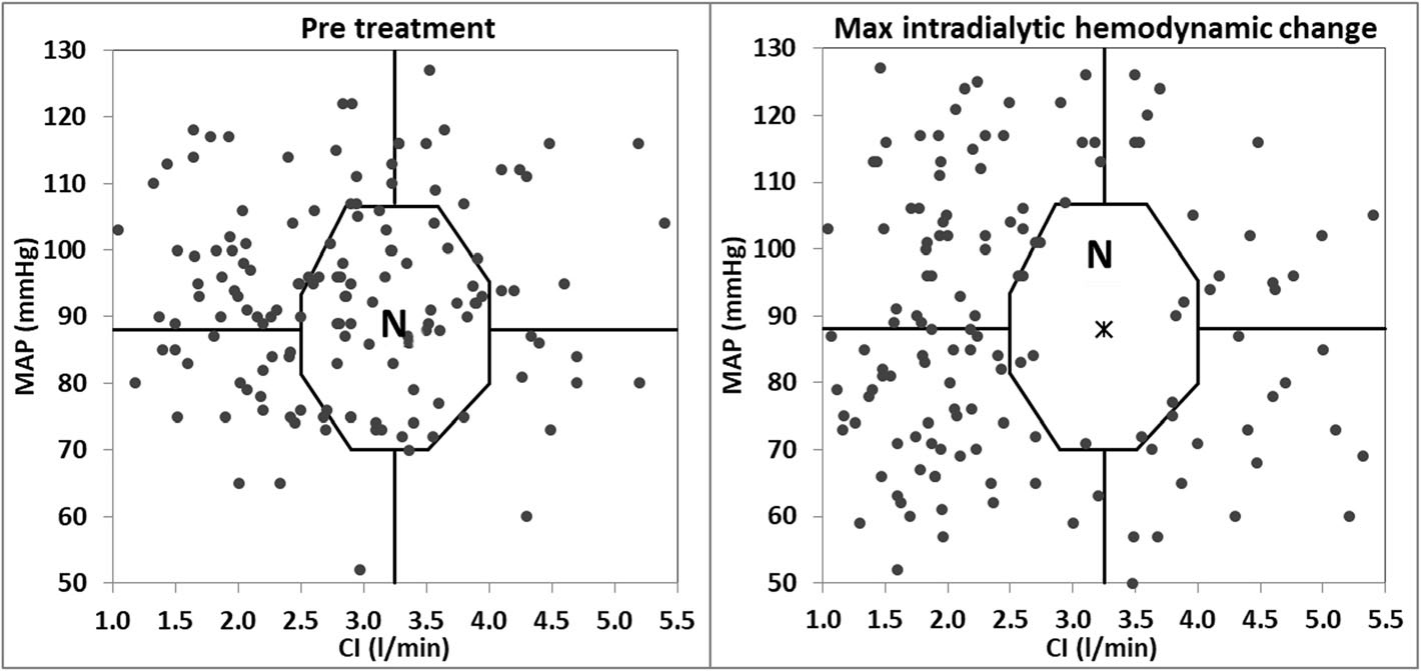
Hemodynamic status at pretreatment and at maximal change from baseline during dialysis. Left panel- patients’ hemodynamic status as represented by their pretreatment cardiac index (CI); Mean arterial pressure (MAP); Right panel- patients’ hemodynamic status at maximal change from baseline (CI;MAP). “N” stands for normal hemodynamics

For this study, each patient underwent a single NICaS monitored HD session. Hemodynamic parameters (including HR, SI, CI, TPRI, and CPI) were measured prior to, during (at intervals of 30–60 min) and 5–10 min after dialysis. Both the HD prescription and the interventions performed during and after HD were independent of the data collected by the system. For a graphic representation of hemodynamic status, a graph with CI on the *x*-axis and MAP on the *y*-axis is shown (Fig. 1). Normal hemodynamic status, as depicted in the center of the figure, was defined by the normal range of MAP and CI (70–105 mmHg with a mean value of 88 mmHg and CI of 2.5–4.0 l/min/m^2^ with a mean value of 3.25 l/min/m^2^ respectively). Normal TPRI and CPI ranges are 1600 < TPRI< 3000 dyn*sec/cm^5^*m^2^ and 0.45 < CPI < 0.85 W/m2 respectively (not shown in the figure for simplification) complete the graphical definition of normal hemodynamics by creating an octagon shape. After establishing the normal range, four abnormal hemodynamic profiles based on the CI, MAP, CPI and TPRI values as follows: *high CPI* (MAP≥88 mmHg and CI ≥ 3.25 l/min/m^2^); *high TPRI* (MAP≥88 mmHg and CI < 3.25 l/min/m^2^); *low CPI* (MAP< 88 mmHg and CI < 3.25 l/min/m^2^); *low TPRI* (MAP< 88 mmHg and CI ≥ 3.25 l/min/m^2^).

The (CI, MAP) values of each patient were plotted at three time intervals: The first was prior to HD initiation- this determined the patient’s profile at baseline. The second was during dialysis by plotting the maximal change in hemodynamics occurring during dialysis which was defined as the (CI, MAP) point with the greatest distances from the center of the graph. The third point was the (CI, MAP) point recorded about 5–10 min after dialysis. These three points noted for each patient allowed graphic representation of the hemodynamic changes occurring during and after dialysis.

### Statistical analysis

Continuous variables are expressed as mean ± SD for normally distributed variables and as median [IQR] for non-normally distributed variables. Categorical variables are expressed as percentages. Analysis of variance was used to test variations between groups. A *p*-value < 0.05 was considered significant. Statistical analysis was performed using IBM SPSS (Version 23), IBM Corp. Armonk, NY) and Microsoft Excel (Microsoft Corp. Redmond WA).

## Results

### Cohort description and site comparison

This analysis includes 144 patients, studied in the USA (*n* = 26), China (*n* = 27), Brazil (*n* = 28), and Israel (*n* = 63). The average age was 67.3 ± 12.1 years with male sex prevalence of 56.3%. As detailed in Table 1, pretreatment systolic and diastolic BPs were 139 ± 23 mmHg and 70 ± 13 mmHg respectively. MAP was 93 ± 15 mmHg, CI 2.9 ± 0.9 L/min/m^2^, TPRI 2963 ± 1318 dyn*Sec/cm^5^*m^2^ and CPI 0.59 ± 0.22 W/m2. Complete medication data were available for 90/144 patients. The majority (58.9%) were prescribed with β-receptor blockers, 53.3% with calcium channel blockers (CCB), 46.7% with angiotensin-converting enzyme inhibitor (ACEi)/ angiotensin receptor blockers (ARB) and 16.7% α-receptor blockers. Additional file 1: Table S1 details the comparison in various indices between the patients included in the four sites. Briefly, patients’ age and the prevalence of diabetes mellitus did not differ between the sites but differences in weight and body-mass-index were recorded with the Chinese patients being the patients with lower body weight in comparison to the other sites. Although differences in the total length of dialysis and in total fluid removal were noted, the ultrafiltration rate did not differ between the sites. Differences between hemodynamic patterns were also noted between the population included in each site.

**Table 1:** Baseline patients’ demographic, clinical and hemodynamic characteristics at baseline. Data are presented as n(%), mean ± SD or median [Q1,Q3]

Index	Valve (*n* = 144)
*Baseline characteristics*	
Age (years)	67.3 ± 12.1
Male	81 (56.3%)
Weight (kg)	72.3 ± 16.3
Body Mass Index (kg/m^2^)	26.8 ± 5.7
Dialysis vintage (months)	24.7 [15.2, 41.7]
Diabetes mellitus	77, (53.5%)
Heart failure	36, (25.0%)
*Baseline hemodynamic parameters*	
Systolic blood pressure (mmHg)	139 ± 23
Diastolic blood pressure (mmHg)	70 ± 13
Mean arterial pressure (mmHg)	93 ± 15
Heart rate (beats per minute)	75 ± 12
Stroke index (mL/m^2^)	38.0 ± 11.3
Cardiac index (L/min/m^2^)	2.85 [2.13, 3.53]
Cardiac power index (W/m^2^)	0.56 [0.42, 0.73]
Total peripheral resistance (dyn*sec/cm^5^*m^2^)	2754 [2071,3398]
*Baseline medications*	*n = 90*
Angiotensin converting enzyme inhibitor/ Angiotensin receptor binder	42 (46.7%)
Calcium channel blocker	48 (53.3%)
β receptor blocker	53 (58.9%)
α receptor blocker	15 (16.7%)
Insulin	9 (10%)

Data are presented as n(%), mean ± SD or median [Q1,Q3]

### Hemodynamic response to dialysis

Figure 1 depicts the hemodynamics of all patients prior to HD (left panel) and at the maximal intra-dialytic hemodynamic change (right panel). As opposed to pretreatment, in which a significant proportion of patients was within the normal range of hemodynamics (35.4%), only a few patients maintained these hemodynamics during dialysis (6.9%). Post-HD partial hemodynamic recovery was observed in most patients with 28.5% returning to the normal hemodynamic range.

### Hemodynamic profiles

**Fig. 2:**
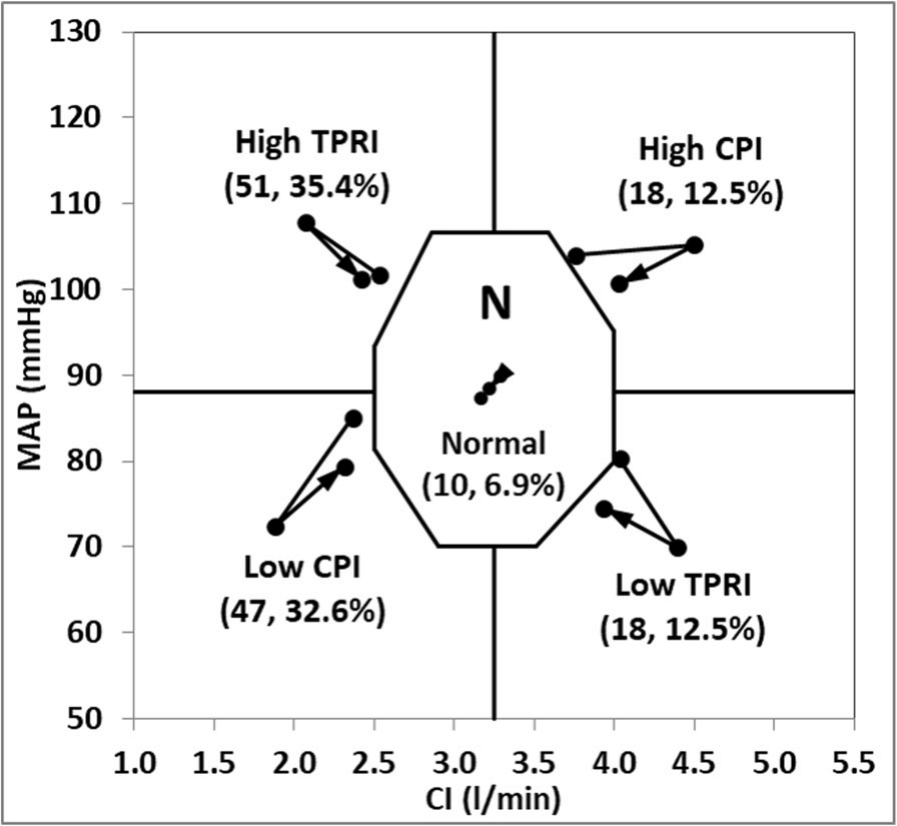
Incidence and trends in hemodynamics for patients with different hemodynamic profiles. Mean cardiac output (CI)/Mean arterial pressure (MAP) pre-dialysis, during maximal change during dialysis and post-dialysis is plotted for each of the five hemodynamic profile. The arrows detail the direction of change between the three time intervals. Normal total peripheral resistance index (TPRI) and cardiac power index (CPI) ranges are 1600 < TPRI< 3000 dyn*sec/cm^− 5^*m^2^ and 0.45 < CPI < 0.85 W/m2; high CPI (MAP≥88 mmHg and CI ≥ 3.25 l/min/m2); high TPRI (MAP≥88 mmHg and CI < 3.25 l/min/m2); low CPI (MAP< 88 mmHg and CI < 3.25 l/min/m2); low TPRI (MAP< 88 mmHg and CI ≥ 3.25 l/min/m2)

The order of frequency of predefined hemodynamic profiles was high TPRI followed by low CPI, high CPI, low TPRI and normal hemodynamics (35.4, 32.6, 12.5,12.5, and 10% respectively). Save for age differences, all the baseline characteristics were similar among the patients in different hemodynamic profiles (Table 2). Total fluid removal and ultrafiltration rates were also similar. Figure 2 details the mean (CI, MAP) of patients in each hemodynamic profile recorded at three-time intervals- pre-HD, during dialysis and post-HD.

**Table 2:** Baseline characteristics, dialysis and hemodynamic data stratified by hemodynamic profile. Data are presented as n(%), mean ± SD or median [Q1,Q3]

Index	Normal *n* = 10 (6.9%)	Low CPI *n* = 47 (32.6%)	Low TPRI *n* = 18 (12.5%)	High CPI n = 18 (12.5%)	High TPRI *n* = 51 (35.4%)	P-value
Demographics						
Male	6 (60.0%)	30 (63.8%)	10 (55.6%)	7 (38.9%)	28 (54.9%)	0.50
Age (y)	68.4 ± 11.7	71.6 ± 12.0	69.1 ± 7.6	61.9 ± 10.8	64.4 ± 13.0	< 0.01
Weight (kg)	73.8 ± 19.1	75.4 ± 15.3	76.4 ± 16.6	66.4 ± 18.7	72.9 ± 15.4	0.22
BMI (kg/m^2^)	27.4 ± 5.3	27.3 ± 5.7	26.2 ± 5.3	25.1 ± 7.1	26.9 ± 5.3	0.67
Diabetes	5 (50.0%)	27 (58.7%)	9 (50.0%)	7 (38.9%)	28 (56.0%)	0.69
Heart failure	1 (10.0%)	14 (29.8%)	6 (33.3%)	3 (16.7%)	12 (23.5%)	0.54
Fluid removal data						
Duration (hh:mm)	3:19 ± 0:53	3:49 ± 0:42	3:40 ± 0:23	3:47 ± 0:35	3:46 ± 0:41	0.28
TFR (ml)	2421 ± 1091	2505 ± 829	2150 ± 836	2008 ± 1112	2553 ± 1039	0.21
UF (ml/kg/h)	10.7 ± 4.6	8.8 ± 3.3	8.9 ± 3.2	8.0 ± 4.3	9.6 ± 4.0	0.36
Pretreatment hemodynamic						
SBP (mmHg)	133 ± 19	130 ± 20	119 ± 13	156 ± 16	150 ± 23	< 0.001
DBP (mmHg)	69 ± 9	63 ± 10	61 ± 11	78 ± 11	78 ± 13	< 0.001
MAP (mmHg)	90 ± 10	85 ± 11	80 ± 10	104 ± 10	102 ± 13	< 0.001
CI (l/min/m^2^)	3.32 [2.85,3.82]	2.31 [1.96,2.80]	4.03 [3.40,4.49]	3.65 [3.23,4.24]	2.61 [1.96,3.08]	< 0.001
CPI (W/m^2^)	0.61 [0.57,0.76]	0.42 0.36,0.51]	0.70 [0.57,0.84]	0.85 [0.71,1.02]	0.56 [0.43,0.72]	< 0.001
TPRI (dyn*sec/cm^5^*m^2^)	2022 [1885,2909]	2985 [2391,3775]	1593 [1433,1858]	2192 [2019.2592]	33,022 [2668,4177]	< 0.001
Intradialytic nadir hemodynamic						
SBP (mmHg)	126 ± 19	111 ± 16	105 ± 17	158 ± 20	157 ± 23	< 0.001
DBP (mmHg)	68 ± 9	54 ± 9	53 ± 9	79 ± 15	84 ± 12	< 0.001
MAP (mmHg)	87 ± 12	72 ± 9	70 ± 10	105 ± 12	108 ± 12	< 0.001
CI (l/min/m^2^)	3.02 [2.68,3.80]	1.81 [1.48,2.07]	4.37 [3.80.5.00]	4.45 [3.7, 4.99]	1.99 [1.77,2.37]	< 0.001
CPI (W/m^2^)	0.59 [0.50,0.70]	0.28 [0.24,0.34]	0.68 [0.56,0.82]	0.99 [0.92,1.13]	0.46 [0.40,0.59]	< 0.001
TPRI (dyn*sec/cm^5*^m^2^)	2210 [1816,2909]	3219 [2795,4211]	1321 [1149,1397]	11,843 [1629,2928]	44,215 [3508,4744]	< 0.001
Post treatment hemodynamic						
SBP (mmHg)	124 ± 16	122 ± 20	110 ± 16	147 ± 25	150 ± 26	< 0.001
DBP (mmHg)	71 ± 10	59 ± 8	58 ± 11	78 ± 12	77 ± 11	< 0.001
MAP (mmHg)	88 ± 10	79 ± 10	75 ± 11	101 ± 13	101 ± 14	< 0.001
CI (l/min/m^2^)	3.07 [2.88,3.70]	2.30 [1.88,2.70]	3.81 [3.35,4.31]	3.75 [3.22,4.62]	2.42 [1.92,2.83])	< 0.001
CPI (W/m^2^)	0.69 [0.57,0.60]	0.40 [0.31,0.50]	0.60 [0.50,0.81]	0.87 [0.76, 1.00]	0.55 [0.44,0.66]	< 0.001
TPRI (dyn*sec/cm^5^*m^2^)	2275 [1807,2730]	2796 [2461,3756]	1494 [1357,1724]	11,992 [1761,2744]	3404 [2812,4243]	< 0.001

*BMI* Body Mass index, *TFR* Total fluid removed, *UF* Ultra filtration rate, *SBP* Systolic blood pressure, *DBP* Diastolic blood pressure, *MAP* Mean arterial pressure, *CI* Cardiac Index, *CPI* Cardiac power index, *TPRI* Total peripheral resistance index, CI, CPI and TPRI are expressed as median [Q1, Q3], all other parameters are expressed as mean ± SD or n(%)

The hemodynamic changes from pre-HD to intra-dialytic and post-HD demonstrates a similar pattern in all four abnormal profiles: after the initial shift from pre-HD to maximal intradialytic hemodynamic change, post-HD hemodynamics tended to return to pre-HD values in all groups. Intradialytic BP decreased in 65 (45.1%) of patients. Of these, 47 (72.3%) were classified as low CPI while 18 (27.7%) were classified as low TPRI. A hypertensive response was recorded in 69 (47.9%) of the patients with 51 (73.9%) demonstrating high TPRI and 18 (26.1%) high CPI.

### Medication use, and outcome stratified by hemodynamic profile

**Fig. 3:**
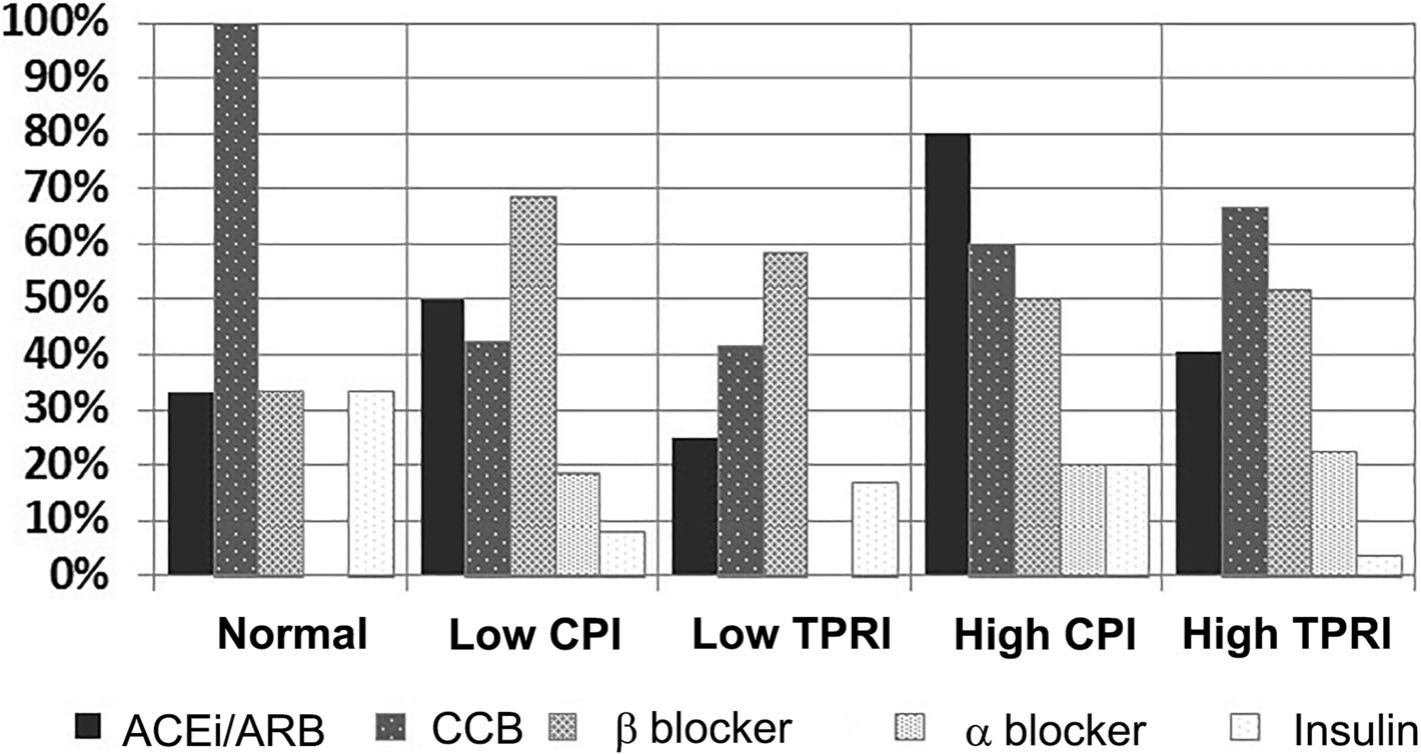
Medication prescription rates for each of the four hemodynamic profiles and those with normal hemodynamics. CPI-cardiac power index; TPRI-total peripheral resistance index; ACEi-angiotensin converting enzyme inhibitor; CCB-calcium channel blocker

High CPI patients had the highest rates of medication use– in 80% either ACEi or ARB and CCB and β receptor blockers were prescribed. (Fig. 3). In low CPI patients, 50% were prescribed with ACEi/ARB and 42.1% CCB. All-cause mortality rate at 12 months of follow-up was 15.3% (*n* = 22). All patients were followed up. The highest incidence of mortality at 12 months follow-up was noted in patients with low CPI (23.4%) and low TPRI (22.3%), followed by high TPRI (9.8%) and high CPI (5.6%). No deaths occurred in patients in the normal hemodynamics group (*p* = 0.06 for intergroup comparison).

## Discussion

The results of this multicenter study demonstrate distinct variability in the hemodynamic response to HD. (1) While a significant proportion of patients demonstrates normal hemodynamics prior to initiation of HD, the procedure induces significant alterations in the hemodynamic status in most patients. (2) These responses can be grouped into 4 separate hemodynamic responses with observed differences in outcome. (3) At the end of dialysis, patients tend to return towards normal hemodynamic status.

Despite many years of experience with HD, the actual hemodynamic changes occurring during this treatment are poorly described despite the dire outcome of patients sustaining IDH [16]. Most data regarding the hemodynamic responses to HD date back to the ‘70s and ‘80s of the twentieth century. These data do not reflect the current practice of shorter, high ultrafiltration rate dialysis [4, 5, 17]. For example, Rouby et al. in 1980 described the hemodynamic response of ten patients undergoing HD and sequential ultrafiltration using an invasive pulmonary artery catheter technique. Similarly to other studies, they recorded decreases in CI and MAP with preserved TPRI. It should be noted that this study excluded patients prescribed with antihypertensive or cardiovascular drugs and those with cardiomyopathies. [5] Beyond being a small scale study, the included population did not reflect a “real-world” HD population in which many patients have multiple co-morbidities [18, 19] and are prescribed with multiple drugs- all of which might potentially impact the hemodynamic response to HD. These gaps in knowledge motivated the initiation of the current study which included all-comers, “real-world” population of HD patients treated with current HD protocols.

**Fig. 4:**
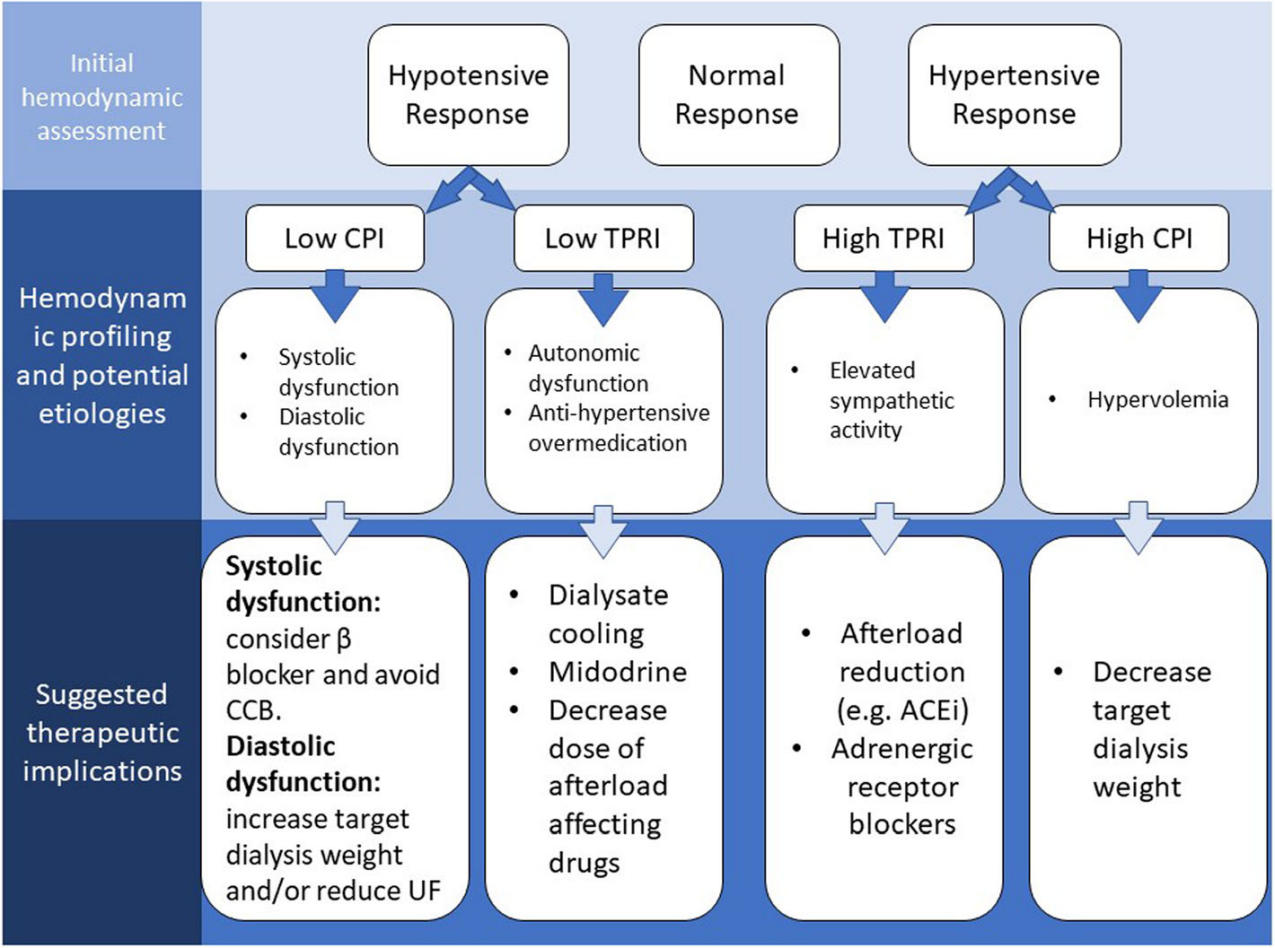
Summary of hemodynamic profiling of HD patients, main etiologies and suggested therapeutic implications. CPI- cardiac power index; TPRI- total peripheral resistance index; CCB- calcium channel blocker; ACEi- angiotensin converting enzyme inhibitor

At present, HD hemodynamics are monitored by intermittent measurements of BP. In the present analysis, in only 6.9% of patients, hemodynamics was preserved during dialysis while in the remainder response was variable. This highlights the need for more relevant assessment tools which would enable a more accurate differentiation of causes for IDH and related clinical problems. IDH is known to be a significant clinical event during dialysis since it is associated with poor long term outcome [1, 20–22]. These and other data clearly indicate that IDH results from two distinct hemodynamic responses- decrease in TPRI or decrease in CPI [15]. Low TPRI can be described as the relative inability of the peripheral vasculature to respond to stimuli for vasoconstriction to compensate for the hypovolemia induced by ultrafiltration. This may result from autonomic dysfunction, probably more common in diabetic patients [23], or overmedication with anti-hypertensive, vasodilator drugs. Low CPI, on the other hand, results mainly from an acute reduction in CO, stemming from either systolic or diastolic cardiac dysfunction. Left ventricular (LV) systolic dysfunction is significantly more prevalent in HD patients compared with the non-HD population [24] probably due to the high prevalence of hypertension and concomitant risk factors for coronary artery disease- the main etiologies for reduced LV function but also due to repetitive stunning induced by IDH as suggested by McIntyre et al [25, 26]. More importantly, diastolic dysfunction (DD), is probably a more common phenomenon than LV systolic dysfunction in HD patients [16]. DD is closely related with left-ventricular hypertrophy both caused by gradual myocardial fibrosis. From a physiological standpoint, HD patients with DD are preload dependent- i.e. their ability to fill the heart is limited and thus if preload is lowered (such as in the case of too rapid fluid removal by ultrafiltration), the CO and CPI will decrease. The ability to differentiate between two separate processes leading to IDH practically should be useful for therapeutic intervention. First, if CPI is reduced during HD, it is important to establish whether systolic or diastolic dysfunction (DD) is present by cardiac evaluation including echocardiography. If systolic dysfunction is diagnosed, certain CCBs should not be prescribed mainly due to their negative inotropic effect [27]. Of note, these drugs were prescribed to approximately 40% of the patients in the low CPI group in the present study. Patients with systolic dysfunction may also benefit from β receptor blockers and ACEi. The former can prolong diastole and the latter reduces afterload. On the other hand, if DD is diagnosed as the etiology for low CPI, assuming that these patients are preload dependent, the target weight goal could be increased and/or the ultrafiltration rate reduced. When low TPRI is diagnosed as the cause for BP decrease, dialysate cooling or use of an adrenergic stimulating agent (e.g. midodrine) may be useful. [28] These patients may also benefit from a decrease in the dose of afterload reducing antihypertensive drugs. The major implication of this ability to differentiate between the two major etiologies of IDH during HD is a paradigm shift toward a more personalized, specific approach to the diagnosis and management of individual episodes of IDH. Similar implications may be relevant for patients demonstrating hypertension during dialysis in which two distinct reactions were demonstrated- either high TPRI or high CPI. Those with high CPI may be hypervolemic and may benefit from a reduction in target dry weight while those with high TPRI, which result from elevated sympathetic over-activity [25, 26] may benefit from the use of pharmacologic afterload reduction (e.g. ACEi) or β-receptor blocker. The sequence of intradialytic analysis interpretation and potential implications is summarized in Fig. 4.

Several limitations to this work are acknowledged: First, this was a relatively small-scale study aimed at establishing the ability of this technique to add clinical knowledge of intradialytic hemodynamics which goes well beyond BP measurement. Thus, the interpretation of these data with regards to the association between certain hemodynamic profiles and mortality should be taken cautiously. Secondly, differences were noted between the baseline characteristics and hemodynamic indices of patients from different sites. Since this was an all-comers study aimed for description of hemodynamic patterns before, during and after dialysis, we believe that the fact that different patients treated in different centers and countries were included truly reflect the diversity of responses seen in dialysis which highlights the need for an online monitoring tool. Thirdly, the maximal change from baseline during dialysis was performed at 30–60 min intervals. It should be acknowledged that hemodynamic changes may have possibly occurred between these intervals, yet we believe that the probability of missing a significant hemodynamic event is relatively low. Finally, this was a proof-of-concept observational study and thus the efficacy of the recommendations given for each of the hemodynamic profile is yet to be determined in prospective interventional studies.

## Conclusions

BP is a poor indicator of hemodynamic status in patients undergoing HD. A more thorough understanding of the different parameters impacting patients’ response to HD is a clear and relevant need. By utilizing a valid non-invasive whole-body impedance device, a relatively complete picture of the patient’s hemodynamic status can be visualized throughout the treatment and a personalized approach to these changes may be applied. This potentially may guide an appropriate response to the varied etiologies of repeated IDH episodes with the reasonable possibility of reversion or prevention of the deleterious outcomes of these episodes.

## Additional file

Additional file 1: **Table S1.** Baseline characteristics and hemodynamic indices grouped by study center. Data are presented as n(%), mean ± SD or median [Q1,Q3]. (DOCX 22 kb)

## Data Availability

The anonymized data is available at: https://drive.google.com/open?id=1wE2HFAIfNZqyDTgeiXTEDchNFFJHDTAV

## Abbreviations

ACEi: Angiotensin converting enzyme inhibitor
ARB: Angiotensin II receptor blocker
BMI: Body mass index
BP: Blood pressure
CCB: Calcium channel blockers
CI: Cardiac index
CO: Cardiac output
DD: Diastolic dysfunction
HD: Hemodialysis
HR: Heart rate
IDH: Intradialytic hypotension
MAP: Mean arterial pressure
SI: Stroke index
SV: Stroke volume
TPRI: Total peripheral resistance index
