# Supplementary material for: Non-invasive hemodynamic profiling of patients undergoing hemodialysis - a multicenter observational cohort study
Source: BMC Nephrol. 2019 Sep 3;20:347. doi: 10.1186/s12882-019-1542-4 (PMC6724365; doi:10.1186/s12882-019-1542-4)
Supplement: Supplementary file 1 — Table S1. Baseline characteristics and hemodynamic indices grouped by study center. Data are presented as n(%), mean ± SD or median [Q1,Q3]. (DOCX 22 kb) [file 12882_2019_1542_MOESM1_ESM.docx]

| **Index** | **Full cohort (n=144)** | **China (n=27)** | **USA (n=26)** | **Brazil (n=28)** | **Israel (n=63)** | | **P-value** |
| --- | --- | --- | --- | --- | --- | --- | --- |
| **Demographics** | | | | | | |  |
| Male | 81 (56.3%) | 12 (44.4%) | 14 (53.8%) | 14 (50.0%) | 40 (63.5%) | | 0.396 |
| Age (years) | 67 ± 12 | 65 ± 12 | 68 ± 8 | 66 ± 12 | 68 ± 14 | | 0.735 |
| Weight (kg) | 72 ± 16 | 62 ± 12 | 71 ± 13 | 77 ± 19 | 75 ± 16 | | <0.001 |
| BMI (kg/m^2^) | 26.8 ± 5.7 | 23.7 ± 3.9 | 27.2 ± 4.3 | 28.2 ± 6.3 | 27.3 ± 6.1 | | 0.019 |
| Diabetes | 72 (52.2%) | 12 (44.4%) | 16 (61.5%) | 15 (53.6%) | 33 (54.1) | | 0.672 |
| **Fluid removal data** | | | | | | | |
| Duration (hh:mm) | 3:44 ± 0:40 | 3:29 ± 0:28 | 3:27 ± 0:50 | 3:40 ± 0:54 | 4:00 ± 0:24 | | <0.001 |
| TFR (ml) | 2410 ± 971 | 1867 ± 891 | 2098 ± 725 | 2571 ± 1149 | 2699 ± 888 | | <0.001 |
| UF (ml/kg/h) | 9.1 ± 3.8 | 8.8 ± 3.7 | 9.8 ± 5.8 | 8.9 ± 3.7 | 9.1 ± 2.8 | | 0.767 |
| **Pre treatment hemodynamic** | | | | | | | |
| SBP (mmHg) | 139 ± 23 | 136 ± 22 | 138 ± 23 | 144 ± 17 | 139 ± 23 | | 0.629 |
| MAP (mmHg) | 93 ± 15 | 90 ± 13 | 93 ± 17 | 102 ± 11 | 90 ± 14 | | 0.017 |
| CI (l/min/m^2^) | 2.9 [2.1, 3.5] | 3.8 [3.6, 3.9] | 3.2 [2.4, 3.6] | 2.8 [2.0,3.2] | 2.4 [2.0, 3.0] | | <0.001 |
| CPI (w/m^2^) | 0.56 [0.42, 0.73] | 0.81 [0.53, 0.89] | 0.58 [0.45, 0.73] | 0.61 [0.46, 0.72] | 0.45 [0.36, 0.63] | | <0.001 |
| TPRI (dyn*sec/cm^2^*m^2^) | 2754 [2071, 3398] | 1969 [1685, 2192] | 2539 [2025, 2909] | 2853 [2494, 4021] | 3022 [3158, 2907] | | <0.001 |
| TBW (%) | 52.9 ± 9.8 | 54.2 ± 8.2 | 52.6 ± 10.2 | 49.6 ± 9.1 | 53.8 ± 10.4 | | 0.287 |
| **Intradialytic nadir hemodynamic** | | | | | | | |
| SBP (mmHg) | 133 ± 30 | 128 ± 30 | 129 ± 23 | 143 ± 25 | 133 ± 31 | | 0.240 |
| MAP (mmHg) | 90 ± 20 | 87 ± 20 | 89 ± 22 | 101± 17 | 87± 20 | | <0.001 |
| CI (l/min/m^2^) | 2.3 [1.8, 3.5] | 3.7 [2.7, 4.6] | 3.4 [2.4, 4.3] | 2.3 [1.9, 2.7] | 1.9 [1.6, 2.2] | | <0.001 |
| CPI (w/m2 [IQR]) | 0.45 [0.33, 0.68] | 0.65 [0.47, 0.92] | 0.46 [0.38, 0.65] | 0.46 [0.38, 0.65] | 0.35 [0.26m 0.48] | | <0.001 |
| TPRI (dyn*sec/cm^2^*m^2^) | 3044 [1958, 4214] | 1644 [1470, 2820] | 2051 [1613, 1403] | 3251 [2855, 4391] | 3586 [2898, 4549] | | <0.001 |
| **Post treatment hemodynamic** | | | | | | | |
| SBP (mmHg) | 134 ± 27 | 125 ± 25 | 133 ± 29 | 137 ± 24 | 135 ± 27 | | 0.295 |
| MAP (mmHg) | 90 ± 16 | 86 ± 15 | 90 ± 19 | 97 ± 14 | 87 ± 16 | | 0.046 |
| CI (l/min/m^2^) | 2.7 [2.1, 3.3] | 3.3 [3.0, 4.1] | 3.4 [2.8, 3.9] | 2.8 [2.2, 3.0] | 2.3 [1.9, 2.7] | | <.001 |
| CPI (w/m^2^) | 0.54 [0.40, 0.69] | 0.60 [0.52, 0.82] | 0.69 [0.54, 0.83] | 0.59 [0.43, 0.71] | 0.44 [0.35, 0.55] | | <0.001 |
| TPRI (dyn*sec/cm^2^*m^2^) | 2710 [1999, 3456] | 1935 [1715, 2340] | 2077 [1629, 2604] | 2877 [2664, 3939] | 3039 [2374, 4002] | | <0.001 |
| TBW (%) | 51.3 ± 10.1% | 52.7 ± 8.6% | 51.3 ± 10.5% | 47.4 ± 9.6% | 51.6 ± 11.3% | | 0.404 |
| **Hemodynamic profiles** |  |  |  |  |  | |  |
| Normal | 6.9% | 7.4% | 15.4% | 10.7% | 1.6% | | 0.099 |
| Low CPI | 32.6% | 18.5% | 11.5% | 21.4% | 52.4% | | <0.01 |
| Low TPRI | 12.5% | 29.6% | 23.1% | 0.0% | 6.3% | | <0.01 |
| High CPI | 12.5% | 25.9% | 19.2% | 10.7% | 4.8% | | 0.027 |
| High TPRI | 35.4% | 18.5% | 30.8% | 57.1% | 34.9% | | 0.023 |
| **Legend**: BMI-Body Mass index; TFR- Total fluid removed; UF- Ultra filtration rate; SBP- Systolic blood pressure; MAP- Mean arterial pressure; CI- Cardiac Index; CPI- Cardiac power index; TPRI- Total peripheral resistance; TBW- Total body water. Data are presented as n(%), mean±SD or median[Q1,Q3]. | | | | | |  |  |
